# Effects of Aspirin on Endothelial Function and Hypertension

**DOI:** 10.1007/s11906-016-0688-8

**Published:** 2016-10-27

**Authors:** Mikhail S. Dzeshka, Alena Shantsila, Gregory Y. H. Lip

**Affiliations:** 1 University of Birmingham Institute of Cardiovascular Sciences, City Hospital, Dudley Road, Birmingham, B18 7QH UK; 2 Grodno State Medical University, Grodno, Belarus; 3 Aalborg Thrombosis Research Unit, Department of Clinical Medicine, Aalborg University, Aalborg, Denmark

**Keywords:** Aspirin, Endothelial function, Arterial hypertension, Cyclooxygenase, Platelets, Inflammation

## Abstract

**Purpose of review:**

Endothelial dysfunction is intimately related to the development of various cardiovascular diseases, including hypertension, and is often used as a target for pharmacological treatment. The scope of this review is to assess effects of aspirin on endothelial function and their clinical implication in arterial hypertension.

**Recent findings:**

Emerging data indicate the role of platelets in the development of vascular inflammation due to the release of proinflammatory mediators, for example, triggered largely by thromboxane. Vascular inflammation further promotes oxidative stress, diminished synthesis of vasodilators, proaggregatory and procoagulant state. These changes translate into vasoconstriction, impaired circulation and thrombotic complications. Aspirin inhibits thromboxane synthesis, abolishes platelets activation and acetylates enzymes switching them to the synthesis of anti-inflammatory substances.

**Summary:**

Aspirin pleiotropic effects have not been fully elucidated yet. In secondary prevention studies, the decrease in cardiovascular events with aspirin outweighs bleeding risks, but this is not the case in primary prevention settings. Ongoing trials will provide more evidence on whether to expand the use of aspirin or stay within current recommendations.

## Introduction

Endothelium is of paramount importance for maintaining homeostasis of cardiovascular system [1]. Healthy endothelium, both in vasculature and heart chambers, is continuously releasing plethora of bioactive substances. Acting in autocrine, paracrine and systemic fashion and participating in regulation of smooth muscle contractions, vascular wall permeability, platelet aggregation, activation of coagulation and fibrinolytic activity, cellular proliferation, as well as in prevention of inflammatory cells adhesion and vascular inflammation. Imbalance between any of above functions is broadly defined as endothelial dysfunction. To the different extent, most cardiovascular diseases are classically attributed to endothelial dysfunction (from atherosclerotic heart disease to rhythm disturbances, e.g. atrial fibrillation) [2–4].

Given that endothelium takes part in regulation of vascular tone, arterial hypertension and endothelial function are reciprocally and intimately associated with each other. However, this association is far beyond simple imbalance between vasodilator and vasoconstrictor release in favour of the latter. Multiple mechanisms are involved in regulation of blood pressure, including the endothelium, kidneys and central regulation [5].

Of note, the endothelium does not only provide short-term effects on vascular tone. Endothelial dysfunction also has chronic long-term consequences, which, in indirect way, eventually have major impact on vascular remodelling and blood pressure regulation. Oxidative stress, i.e. production of free radicals outweighing their scavenging, is one of the systemic pathological processes, related to endothelium. Inflammation in the vascular wall is another example, synthesis of pro-inflammatory cytokines and recruitment of inflammatory cells, with hypertension being considered even as inflammatory disease now [5, 6].

Endothelial function has been extensively used as putative target for pharmacological correction with drugs inhibiting renin-angiotensin-aldosterone system, statins, antioxidants and so on, but less attention was paid to vascular effects of aspirin (i.e. acetylsalicylic acid) given its primary anti-aggregatory mechanism of action. However, with the emerging evidence on role of platelets in inflammatory reactions and immunomodulation, platelet inhibition with aspirin has been found to also elicit also anti-inflammatory effects [7, 8, 9•, 10]. Moreover, aspirin was found to work as acetylating agent with a range of beneficial effects on vascular endothelium beyond platelet inhibition.

With regard to the possible effects of aspirin on blood pressure and management of arterial hypertension, these will be discussed in this review article.

### Brief Overview of Aspirin Pharmacology: Inhibition of Cyclooxygenase-1

Aspirin has been used for years as analgesic, antipyretic and anti-inflammatory drug due to non-selective cyclooxygenase (COX)-1 and COX-2 inhibition (historically at high doses). Later, the major indication for aspirin shift to prevention of thrombotic cardiovascular complications as anti-platelet drug via predominant COX-1 inhibition within platelets, achievable at low doses.

Cyclooxygenases are present in the endothelial cells and tissues in two isoforms: COX-1 is considered to be a constitutively expressed enzyme, assuring physiological functions while COX-2 isoform is thought to carry potential for inducibility, but it is expressed constitutively at lower levels too. Cyclooxygenases are also defined as prostaglandin endoperoxide synthases or prostaglandin (Pg) G/H synthases since first PgG_2_ is synthesised from the arachidonic acid by incorporation of oxygen molecules, then PgH_2_ is formed by reduction of PgG_2_. PgH_2_ is a substrate for enzymes, which via isomerisation, reduction or other transformations produce range of prostanoids. For example, thromboxane A_2_ (TxA_2_) is synthesised by TxA_2_ synthase (TXAS) and prostacyclin (PgI_2_) is synthesised by prostacyclin synthase (PGIS) [11].

**Fig. 1:**
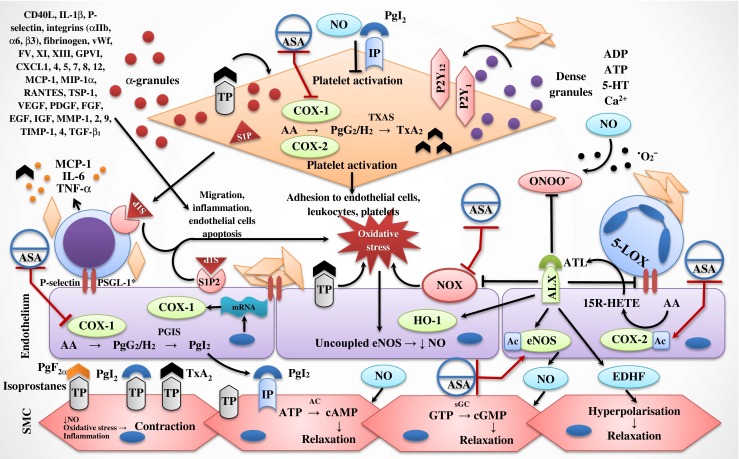
Influence of aspirin on endothelial function. * Various receptors participate in cellular interactions. *15R-HETE*, 15-Hydroxyeicosatetraenoic acid; *5-HT*, serotonin; *5-LOX*, 5-lipooxygenase; *AA*, arachidonic acid; *AC*, adenylate cyclase; *IP*, prostacyclin receptor; *ADP*, adenosine diphosphate; *ALX*, ATL receptor; *ASA*, acetyl salicylic acid; *Ac*, acetyl group; *ATL*, aspirin-triggered 15-epi-lipoxin A_4_; *ATP*, adenosine triphosphate; *cAMP*, cyclic adenosine monophosphate; *cGMP*, cyclic guanosine monophosphate; *COX*, cyclooxygenase; *CXCL1*, 4, 5, 7, 8, 12, chemokine (C-X-C motif) ligand 1, 4, 5, 7, 8 and 12, respectively; *EDHF*, endothelium-derived hyperpolarising factor; *EGF*, epidermal growth factor; *eNOS*, endothelial nitric oxide synthase; *FV*, *XI*, *XIII*, coagulation factors V, XI, XIII, respectively; *FGF*, fibroblast growth factor; *GPVI*, collagen receptor; *GTP*, guanosine triphosphate; *HO-1*, heme oxygenase 1; *IGF*, insulin-like growth factor; *IL-1β*, interleukin-1β; *IL-6*, interleukin-6; *MCP-1*, monocyte chemoattractant protein 1; *MIP-1α*, macrophage inflammatory protein 1α; *MMP-1, 2, 9*, matrix metalloproteinase 1, 2 and 9, respectively; *mRNA*, matrix ribonucleic acid; *NO*, nitric oxide; *NOX*, nicotinamide adenine dinucleotide phosphate-oxidase; *PDGF*, platelet-derived growth factor; *PgG*_*2*_ */H*_*2*_, prostaglandin G_2_/H_2_; *PgI*_*2*_, prostacyclin; *PGIS*, prostacyclin synthase; *PSGL-1*, P-selectin glycoprotein ligand 1; *RANTES*, regulated on activation, normal T Cell expressed and secreted; *S1P*, phingosine-1-phosphate; *S1P2*, S1P receptor 2; *sGC*, soluble guanylate cyclase; *SMC*, smooth muscle cell; *TGF-β*_*1*_, transforming growth factor beta 1; *TIMP-1*, *4*, tissue inhibitor of MMP 1 and 4, respectively; *TNF-α*, tumour necrosis factor α; *TP*, thromboxane prostanoid receptor; *TSP-1*, thrombospondin 1; *TxA*_*2*_, thromboxane A_2_; *TXAS*, thromboxane A_2_ synthase; *VEGF*, vascular endothelial growth factor; *vWF*, von Willebrand factor

These inhibitory effects of aspirin are determined by presence of acetyl group that leads to acetylation of serine hydroxyl group at position 529 in COX-1 with eventual irreversible inhibition of the enzyme activity due to its inability to bind to substrate, arachidonic acid. Inhibition of platelet-dependent synthesis of TxA_2_ is accompanied by diminished release of platelets granules, numerous chemokines, growth factors and coagulation factors (Fig. 1) [7, 9•].

Many other ‘pleiotropic’ favourable effects of aspirin, which are not directly related to inhibition of TxA_2_ synthesis, are attributed to acetylation of target proteins. Their total number is far beyond half a thousand molecular targets, including transcription factors, enzymes, genes, metabolites and so on [12, 13•, 14].

The maximal plasma concentration of aspirin reaches 1 mg/L within half an hour for aspirin 100 mg taken orally and 3 mg/L for aspirin 300 mg taken orally. These doses do not inhibition of COX-2 with COX-1 inhibition being the net pharmacological effect [8, 15]. Because of irreversible action of aspirin (i.e. acetylation), duration of its effects is determined by target protein resynthesis, that in case of mature platelets lacking nuclei is equal to their lifetime (approximately 1 week). New platelets are needed in circulation to recover TxA_2_ synthesis. The endothelial effects of aspirin are more short lived as only few hours are required for endothelial cells to restore their capacity for COX-1 regeneration [8].

### Thromboxane A_2_ and Prostacyclin in Platelets and Vasculature

Thromboxane A_2_ is synthesised via common pathway with other prostanoids and is largely deposited in platelets, although the non-platelet sources (e.g. leukocytes, endothelial cells) exist (Fig. 1). Effects of TxA_2_ are mainly local and realised in autocrine (i.e. platelet activation) and paracrine (e.g. on endothelial cells, leukocytes and so on) manner rather than systemic [8, 10, 11, 16, 17]. Multiple biological effects of TxA_2_ are due to binding of TxA_2_ prostanoid (TP) receptors, which are widely expressed in human body, including platelets, endothelium and smooth muscle cells in vasculature and myocardium [16]. Genetic polymorphism of TP receptors may result in their hypo- or hyperreactivity [18]. Expression of TP receptors on the cellular surface is enhanced in various cardiovascular diseases. For example, in vessels affected by atherosclerosis, there is a 3-fold increase of TP receptors density observed [19].

Activation of TP receptors on platelets leads to platelet activation and further amplification of TxA_2_ synthesis and release. Moreover, platelets α-granule content is released to the blood flow that is represented by a multitude of biologically active molecules including coagulation, proinflammatory and growth factors, adhesive receptors and so on (Fig. 1). Following their release, these factors induce leukocytes recruitment, formation of heterotypic aggregates (platelets–leukocytes), promote inflammation, oxidative stress and remodelling of the vascular wall [7, 9•, 20–22].

Oxidative stress is prominent in cardiovascular diseases including hypertension, and it typically leads to reduced NO bioavailability and NO-dependent relaxation (Fig. 1) [23, 24]. Oxidative stress stimulates TxA_2_ pathway by TXAS upregulation and reduced degradation of the immature form of TP receptors, thus stabilising them; vice versa, continuous signalling via TP receptors leads to downstream generation of more reactive oxygen species (ROS) [25–27]. Angiotensin II, the major effector of the renin-angiotensin system not only promotes hypertension, myocardial and vascular remodelling, but it also contributes to activation of TxA_2_ synthesis and increased expression of TP receptors either directly or through nicotinamide adenine dinucleotide phosphate oxidase (NOX) activation [28, 29].

Endothelial nitric oxide synthase (eNOS) uncoupling (via oxidation of eNOS cofactor tetrahydrobiopterin) and diminished NO bioavailability are other well-recognised consequences of oxidative stress in endothelium (Fig. 1). Essentially, these processes lead to reduction of its vasodilation, anti-aggregatory and anti-inflammatory effects and exaggeration of above described changes [27]. Instead of counterbalancing of vasoconstriction, platelet activation and other pathological effects of TxA_2_, NO when exposed to ROS, results in peroxynitrite formation. This was found to upregulate TxA_2_ synthesis and nitration of PGIS and promote platelet activation [30, 31]. Platelet activation is also associated with a release of thrombospondin-1 from α-granules that was shown to diminish NO-dependent vasodilation in arteries via induction of ROS [32].

Thromboxane A_2_-triggered NOX also upregulates phosphodiesterase type 4, which leads to hydrolysis of a downstream mediator of prostacyclin, cyclic adenosine monophosphate (cAMP), reducing its favourable vasoprotective and vasodilatory effects [33]. At a background of oxidative stress 8-isoprostane (PgF_2α_) is synthesised in increased amount and may serve as TP receptor ligand, causing similar to TxA_2_ downstream effects [34]. Selective blockade of NOX, on the contrary, is associated with decrease in TxA_2_, and it improves vasodilatory response in arteries [35].

Apart from ROS-mediated decrease in NO, stimulation of TP receptors also inhibits endothelial NO production via direct suppression of eNOS phosphorylation [36]. This relationship appears to be reciprocal, as inhibition of NO production, e.g. with L-NAME (N^G^-methyl-l-arginine acetate ester), activates aggregation of platelets [37]. Consequently, NO within platelets is involved in regulation of platelet activation and aggregation (Fig. 1). The mechanisms involved are not entirely clear, and they are at least partly mediated by a NO-dependent release of calcium from the platelet dense granules. Reduction in NO levels causes accumulation of calcium in platelets cytoplasm. This triggers TxA_2_ synthesis from the arachidonic acid located within the platelet membrane phospholipids. Interestingly, Banerjee et al. showed that decreased NO synthesis in platelets was a convergent point in platelet activation pathway irrespectively of an activating agent via various platelet receptors (i.e. adenosine diphosphate, collagen, thrombin or epinephrine) [38].

Endothelium-dependent hyperpolarisation, another important mechanism of vasodilation and regulation of the blood flow, is also interfered with TxA_2_ due to its involvement in modulation of Ca^2+^-activated potassium channels [39]. Moreover, it impairs signal propagation through gap junctions within endothelial layer and smooth muscle cells that affects endothelium dependent hyperpolarisation and vasodilation (Fig. 1) [39].

In line with NO-dependent mechanisms and endothelium-dependent hyperpolarisation, prostacyclin counteracts the vasocontracting factors, like as TxA_2_ and other prostanoids, endothelin I, angiotensin II, etc. Prostacyclin can be synthesised both in endothelial cells and vascular smooth muscle cells, and it is the most abundant prostaglandin among other prostanoids. Similarly to TxA_2_, prostacyclin works locally, and then metabolised to 6-keto-PgF1α. Prostacyclin effects are realised via prostacyclin receptors (IP), which are also have widely distributed in tissues (Fig. 1) [11, 40, 41].

There are also two competing points of view whether COX-1 or COX-2 is the main isotype contributing to prostacyclin synthesis [11, 40, 42, 43]. This question has been extensively discussed, since a decrease in prostacyclin levels has long been considered as the main cause of adverse cardiovascular events associated with COX-2 selective inhibitors [44]. Indeed, selective COX-2 depletion in vascular smooth muscle cells and endothelial cell in mice decreased prostacyclin levels, which was associated with blood pressure elevation and accelerated atherogenesis [45]. However, recent data favour COX-1, as the source of prostacyclin synthesis in vasculature both under basal conditions and stimulation with shear stress or pharmacologically [43, 46, 47].

These data may provide some insights into the mechanisms of hypertension. Simplistically, hypertension occurs when endothelium-dependent vasoconstriction outweighs vasorelaxation, due to either decreased production of endothelium-derived relaxing factors or increased production of endothelium-derived contractile factors, or both [5]. Obviously, pathogenesis of hypertension is complex and this is only one of the multiple pathways involved.

Interplay between mediators, receptors and cellular environment is also complex. This can be illustrated by the prostacyclin effects on vasculature. COX-1 is likely to be the main source of prostacyclin synthesis; however, COX-2 is likely to input towards the prostacyclin pool, particularly in various conditions known to be associated with endothelial dysfunction, e.g. arterial hypertension, atherosclerosis, diabetes mellitus and aging [43, 48, 49]. Also patterns of enzymes and receptors expression may vary, depending on setting.

In terms of signalling via TxA_2_ receptors and intermediate metabolite in prostanoi synthesis, PgH_2_ were considered as major drivers of endothelium-dependent contraction, while NO and prostacyclin as potent relaxing factors. However, prostacyclin was yielded to serve as a contracting agent as well, when binding to TP receptors rather than to IP receptors (Fig. 1) [40, 41]. This appears to happen when IP receptors are getting dysfunctional, e.g. in cardiovascular diseases associated with endothelial dysfunction. Compensatory increase in prostacyclin synthesis in these settings (previously considered as protective mechanism) [50] results in more prostacyclin bound to TP receptors [41, 51]. For example, Liu et al. observed contraction of mice aorta, the effect that was eliminated either at a receptor level (via inhibition of TP receptors) or by reduction of prostacyclin synthesis via COX-1 knockout at a background of low TxA_2_ synthesised [52].

Reduced NO release further exaggerates the imbalance [53]. Under physiological conditions, when sufficient NO is available, prostacyclin produces IP receptors-mediated vasodilation. In contrast, when NO availability is low, signalling via TP receptors is triggered by prostacyclin [40, 41, 43]. Given that expression of eNOS in hypertension in endothelium is reduced, prostacyclin is prone to trigger signalling via TP receptors [49].

### Thromboxane A_2_ Inhibition: Cyclooxygenase-1 Versus Target-Specific Drugs

Platelet aggregation is part of maintenance of haemostasis and homeostasis. Nonetheless, in cardiovascular diseases, linked to endothelial damage, platelet functioning exceeds physiological range and risk of thrombotic complications increases [54]. In clinical practice, aspirin is the most commonly used antiplatelet agent for prevention of adverse events in patients with cardiovascular or cerebrovascular disease [55]. Preventive effect is achieved via inhibition of platelet activation and aggregation due to inhibition of TxA_2_ synthesis. Apparently, one can also anticipate breaking the vicious cycle of TxA_2_-mediated platelet activation, oxidative stress, vascular inflammation, eNOS uncoupling and reduced NO bioavailability with TxA_2_ inhibition. Resulting effects are essentially beneficial for vascular function irrespectively of the nature of disease, e.g. coronary artery disease, hypertension, or arrhythmia.

Indeed, taking into account consequences of activations of TP receptors, their inhibition could be clinically beneficial. Several drugs were developed to avoid side effects associated with COX-1 inhibition, but to retain beneficial effects of TP signalling interruption [56, 57]. A selective inhibitor of TXAS synthase and TP receptor antagonist (BM-573) was tested in apolipoprotein E knockout mice, a model of atherosclerosis associated with reduced endothelium-derived relaxation and NO bioavailability, enhanced oxidative stress and blood pressure elevation. The treatment led to improvement in all of the above parameters [56]. Reduction in blood pressure and abolished atherosclerosis progression were also observed in other experimental studies of TP antagonists [28, 58, 59].

Terutroban (S18886) is perhaps the best known TP receptor antagonist. In animal studies, terutroban showed ability to reduce NOX upregulation and ROS production [60], improve endothelial function [36] and attenuate renal damage in hypertension [61]. In spontaneously hypertensive stroke-prone rats, the use of terutroban prevented cell proliferation in the vessel media, abolished accumulation of collagen and fibronectin in the vascular wall and inhibited expression of heat shock protein-47, MCP-1 and transforming growth factor 1β [62, 63].

Although in some experiments effects of terutroban on inflammation and endothelial function even outweighed effects of aspirin [63], positive effects of TP receptors blockade obtained in animal models did not translate into better outcomes in humans [64]. In the PERFORM trial (Prevention of cerebrovascular and cardiovascular Events of ischaemic origin with teRutroban in patients with a history oF ischaemic strOke or tRansient ischaeMic attack), there was a similar rate of primary end-point of fatal or non-fatal ischaemic stroke, fatal or non-fatal myocardial infarction, or other vascular death observed in terutroban and aspirin groups (11 vs. 11 %, hazard ratio (HR) 1.02, 95 % confidence interval (CI) 0.94–1.12) and increased risk of minor bleeding in terutroban arm (12 vs. 11 %, HR 1.11, 95 % CI 1.02–1.21) [64]. The trial was stopped prematurely. There was also no difference in carotid atherosclerosis progression, assessed by carotid intima-media thickness measurements and carotid plaques between the two treatment groups [65]. Nonetheless, despite the lack of clinical success so far, selective inhibition of TxA_2_, either by synthesis or TP receptors or both, remains one of attractive pharmacological targets in cardiology [56, 57].

### Aspirin and Endothelial Function: Beyond Thromboxane A_2_ Inhibition

What makes the difference between aspirin and selective TxA_2_ inhibition given controversies between animal and bedside data? One explanation is that dual inhibition, TXAS and TP receptors is required because the latter can be activated by other substances, e.g. isoprostanes [56]. Obviously, the more pathways of platelets activation are blocked, the higher effectiveness of treatment is expected in relation to both clinical outcomes and endothelial function [66, 67]. Also, aspirin has a plethora of favourable vascular effects in addition to modulation of the COX-1-dependent TxA_2_ synthesis and platelet activation, which will be discussed below. Noteworthy, decrease in prostacyclin synthesis in endothelium, due to COX-1 inhibition, was thought to be an unfavourable effect of aspirin, now in light of emerging role of prostacyclin in TP receptor signalling is considered to be advantageous [48]. It was also discovered that IP and TP receptors were capable of formation of heterodimeric receptor complex. Within such complex biological downstream effects of TP receptors can shift towards those realised via IP receptors stimulation [68].

#### Endothelial Nitric Oxide Synthase Acetylation

Aspirin was found to acetylate lysine of eNOS, which evokes activation of its enzymatic activity, i.e. NO synthesis, release and bioavailability of NO not only in endothelial cells, but also in platelets (Fig. 1). Moreover, this effect appeared to be independent of COX-1 inhibition and TxA_2_ production [69–72]. Obviously, in platelets pre-treated with aspirin, TxA_2_ level is reduced; thus, their activation is prevented by modulation of both pathways, and downstream decrease in platelet-mediated inflammation in vascular wall can be expected [38, 73].

Two small clinical trials assessed the effect of various doses of aspirin, ranging 81 to 1300 mg, on NO production in patients with metabolic syndrome and coronary artery disease. NO production was indirectly assessed based on levels of heme-oxygenase-1 (HO-1), which is known to be upregulated with increased NO production, and asymmetrical dimethylarginine (ADMA), that serves as eNOS inhibitor. These biomarkers were measured at baseline and after 12 weeks of the treatment. In both primary and secondary prevention cohorts, aspirin increase in HO-1 and decrease in ADMA indicate its ability to increase NO production [74, 75].

#### Aspirin-Triggered Lipoxins and Resolvins

Lipoxins are a type of lipid mediators generated from arachidonic acid. Following aspirin intake, COX-2 is acetylated that switches its enzymatic activity from a prostaglandin endoperoxide synthase to a lipoxygenase pathway (Fig. 1). First, intermediate 15(R)-hydroxyeicosatetraenoic acid is synthesised, then it is converted to ATL by 5-, 12-, or 15-lipoxygenases in various cell types (e.g. endothelial cells, platelets and leukocytes) to 15R-epimers of intrinsic lipoxin A4 and B4, defined as 15-epi-lipoxins or aspirin-triggered lipoxins (ATL) [76, 77].

Aspirin-triggered lipoxins are considered to be more potent than intrinsic lipoxins with effects mediated via appropriate receptor lipoxin A4 receptor (ALX) / formyl peptide receptor (FPR2) with high affinity to it [76, 77]. Aspirin-triggered lipoxins are capable of reduction of NOX-mediated endothelial production of ROS via suppression of redox-sensitive activation of the transcriptional factor nuclear factor-kappa B, induced by either angiotensin II, tumour necrosis factor-α, or thrombin [78]. Aspirin-triggered lipoxins also block platelet-derived growth factor-stimulated proliferation and migration of smooth muscle cells in vasculature [79]. ATL were shown to reduce adhesion of human leukocytes to endothelial cells, reducing inflammation within the vascular wall [80, 81]. Given the role platelets play in regulation of leukocytes adhesion to vascular endothelium, lipoxins released by platelet–leukocyte aggregates control leukocyte activation and adhesion and reduce damage to the vascular wall [82]. Noteworthy, ATL levels were found to be reduced in patients with atherosclerotic lesions, particularly in patients with advanced atherosclerosis [79].

Resolvins (resolution phase interaction products) represent another group of substances with potent anti-inflammatory properties. Resolvins are synthesised from docosohexaenoic and eicosopentaenoic omega-3 polyunsaturated fatty acids (PUFA); hence, PUFA supplementation was found to restore resolvins level if decreased in cardiovascular disease [83].

For example, resolvin E1 (RvE1) is generated by the transformation of 18R-hydro (peroxy)-eicosapentaenoic acid, which in turn is synthesised by the aspirin-acetylated COX-2 in endothelium. Resolvin E1 inhibits transmigration and infiltration of polymorphonuclear leukocytes in vascular wall as well as formation of platelet aggregates [84]. Significant decrease of expression of pro-inflammatory cytokines and adhesion molecules, increase in RvE1 level and, interestingly, decrease in blood pressure were observed in mice treated with fish oil (as source of PUFA) and aspirin [85].

#### Sphingosine-1 Phosphate

Lysosphingolipid sphingosine-1 phosphate (S1P) is another mediator released from platelets in large quantities upon activation, during thrombus formation and inflammation. Given that the S1P release is promoted by TxA_2_, aspirin indirectly inhibits S1P signalling (Fig. 1). Sphingosine-1 phosphate is produced via two isoforms of sphingosine kinase (SphK), of which SphK2 is predominant in platelets, and then binds to S1P receptors on endothelial cells and smooth muscle cells [86].

Circulating S1P may confer protective signalling for vasculature by taking part in maintenance of endothelial layer integrity, reduction in expression of adhesion molecules in endothelium and inhibition of leukocyte adhesion to the endothelium, and increase NO production (mostly via S1P1 receptor). On the contrary, opposing effects are triggered by high levels of S1P released locally from the activated platelets (via S1P2 receptor) [87].

### Clinical Implications of Aspirin Use in Hypertension

#### Impact of Aspirin on Vasculature and Blood Pressure: Bedside Data

Few studies addressed impact of aspirin on vascular function and blood pressure in patients with arterial hypertension. Moreover, the studies were generally small and heterogeneous in term of the studies population, concomitant drugs use, aspirin dose, duration of treatment and generally had small numbers. For example, Pietri et al. assessed effect of 160 mg of aspirin administered for 2 weeks, on blood pressure and parameters of arterial stiffness in a small group, of untreated patients with mild hypertension. They observed 0.5 m/s reduction in pulse wave velocity in the aspirin arm of the study (which they reasonably attributed to endothelial function and vascular tone), and there were no changes found in placebo arm. There was no significant decrease in blood pressure as well [88]. Another study, with similar aspirin dosing and duration of treatment, showed improvement in flow-mediated dilation and decrease in C-reactive protein and intercellular adhesion molecules level with aspirin. However, due to the study design, it was impossible to reliably separate effects of aspirin from effects of concomitant treatments [89]. In another study, aspirin therapy resulted in improvement of flow-mediated dilation and blood pressure reduction, when combined with statins, while no significant dynamics was observed on aspirin monotherapy [90].

The impact of aspirin on blood pressure was found to depend on the time of administration and also to differ in males and females. Hermida et al. performed a series of studies that addressed time-dependent effect of low-dose aspirin (100 mg) administration. Interestingly, a 3-month course of treatment resulted in a minor but significant reduction of ambulatory blood pressure when patients received aspirin at bedtime rather than at awakening, both in mild hypertension and pre-hypertension states [91–94]. The effect was consistent across patients subgroups, but particularly pronounced in females and non-dippers [93, 94].

Overall, robust clinical data on effects of aspirin on vascular function and blood pressure control are scarce, which prevents reliable conclusion on clinical significance of these effects.

#### Prevention of Cardiovascular and Cerebrovascular Events

Despite high blood pressure values being the major cause of vascular complications per se, duration of hypertension, particularly when poorly controlled, is associated with ‘silent’ endothelial damage that in turn hastens atherosclerosis progression [1, 4]. Thus, patients with no clinically apparent coronary artery disease may have their first manifestation of CAD as acute one, e.g. acute coronary syndrome [54].

Despite multiple effects of aspirin that in theory can reduce blood pressure, it is not clinically used for blood pressure lowering. However, given that in management of patients with arterial hypertension, prevention of adverse cardiovascular events is crucial, many hypertensive patients use the agent. Aspirin has a large body of evidence favouring its use for the secondary prevention, but use of aspirin for primary prevention remains controversial. Recent European guidelines on cardiovascular disease prevention did not support prophylactic use of aspirin in individuals without established cardiovascular disease because the risk of major bleeding outweighs the minor decrease in rate of major adverse cardiac events [55]. However, European guidelines for the management of arterial hypertension suggested consideration of aspirin use for primary prevention in patient with high cardiovascular risk or reduced kidney function based on more balanced risk-benefit profile in these categories of patients [95].

The US Preventive Services Task Force has also recently updated recommendations on the use of aspirin for the primary prevention of cardiovascular disease and colorectal cancer. Low-dose aspirin is now supported in men and women aged 50 to 59 years who have a predicted risk for myocardial infarction or stroke of at least 10 % over 10 years, with no elevated bleeding risk, and willing to take aspirin within 10 years or longer. In patients aged 60 to 69 years, a decision has to be made on an individual basis. Other age groups were omitted in the document because of the lack of evidence [96].

Broadly, similar principles were incorporated in the recommendations for antiplatelet treatment for primary prevention of cardiovascular disease in the UK. Age over 50 years with a high cardiovascular risk, defined as 10 years risk of greater than 20 %, or reduced renal function (e.g. estimated glomerular filtration rate less than 45 mL/min/1.73 m^2^) are the clinical scenarios, when aspirin treatment in patients with hypertension can be recommended [97].

There is also controversy in relation to aspirin use in primary prevention settings in patients with diabetes, which is closely linked to other cardiovascular risk factors and cardiovascular morbidity, including hypertension and coronary artery disease. Diabetes is known to be associated with persistent TxA_2_-dependent platelet activation [98]. In diabetic patients, aspirin use showed a significant 10 % reduction of risk of major adverse cardiovascular events, with no effect on myocardial infarction, stroke, cardiovascular or all-cause mortality, and a trend towards higher rate of gastrointestinal bleeds [99].

Despite relation between hazards and benefits of aspirin for primary prevention remained broadly stable with adding new clinical trials to meta-analyses (Table 1), update for evidence is still needed at least because of the following reasons. Widespread use of statins for primary prevention needs to be accounted for as well improving control of cardiovascular risk factors, e.g. hypertension itself, smoking and obesity. These factors can modulate the overall net benefits of aspirin for primary prevention, and they need to be accounted for future analyses.

**Table 1:** Overview of meta-analyses and systematic reviews on primary prevention of cardiovascular events with aspirin over past 10 years

Ref.	All-cause mortality	CV mortality	Stroke mortality	MACE	Non-fatal MI	Stroke	Ischaemic stroke	Haemorrhagic stroke	GI bleeding	Major bleeding
Bartolucci et al. [100]	0.935 (0.87-1.00)	0.893 (0.72-1.10)	NA	0.852 (0.79-0.92)	0.755 (0.67-0.85)	0.945 (0.84-1.06)	NA	NA	NA	NA
Antithrombotic Trialists’ Collaboration [101]	NA	0.97 (0.87–1.09)	1.21 (0 · 84–1.74)	0.88 (0 · 82–0.94)	0.77 (0 · 67–0.89)	0.95 (0.85–1.06)	0.86 (0.74-1.00)	1.32 (1.00–1.75)	NA	1.54 (1.30–1.82)^a^
Bartolucci et al. [102]	0.945 (0.881-1.014)	0.956 (0.799-1.143)	NA	0.865 (0.804-0.930)	0.813 (0.667-0.992)	0.919 (0.828-1.021)	NA	NA	NA	NA
Raju et al. [103]	0.94 (0.88-1.00)	0.96 (0.84-1.09)	NA	0.88 (0.83-0.94)	0.83 (0.69-1.00)	0.93 (0.82-1.05)	0.86 (0.75-0.98)	1.36 (1.01-1.82)	1.37 (1.15-1.62)	1.66 (1.41-1.95)
Raju et al. [104]	0.94 (0.89-1.00)	0.95 (0.84-1.07)	NA	0.89 (0.82-0.97)	0.80 (0.64-0.99)	0.94 (0.84-1.06)	NA	1.43 (1.10-1.86)	1.64 (1.30-2.07)	1.69 (1.43-1.98)
Guirguis-Blake et al. [105•]	0.94 (0.89–0.99)	0.94 (0.86–1.03)	NA	NA	0.78 (0.71-0.87)	0.95 (0.85-1.06)	NA	NA	NA	NA
Whitlock et al. [106•]	NA	NA	NA	NA	NA	NA	NA	1.33 (1.03-1.71)	1.59 (1.32-1.91)	1.55 (1.48-1.63)

*CV* cardiovascular, *GI* gastrointestinal, *MACE* major adverse cardiovascular events, *NA* not applicable

^a^Extracranial bleeding, mostly gastrointestinal.

#### Ongoing Trials

There are several on-going trials on the utility of aspirin in primary prevention settings. The ARRIVE (Aspirin to Reduce Risk of Initial Vascular Events) study aims to evaluate the efficacy and tolerability of 100 mg enteric-coated aspirin compared to placebo in patients with no history of established cardiovascular disease and moderate risk of major coronary heart disease events for the prevention of cardiovascular disease events, including myocardial infarction, unstable angina, stroke or transient ischaemic attack, as well as cardiovascular death [107]. In the ASPREE (Aspirin in Reducing Events in the Elderly) trial effects of 100 mg enteric-coated aspirin on the composite primary endpoint, defined as ‘disability-free life’, including onset of dementia, all-cause mortality, or persistent disability in at least one of the Katz Activities are assessed in individuals free of dementia, disability and cardiovascular disease [108].

There are also two ongoing trials, addressing aspirin for primary prevention in diabetic patients, who are known to be particularly prone to develop atherosclerosis and coronary artery disease, but based on current guidelines should not take aspirin for primary prevention [55]. The ACCEPT-D (Aspirin and Simvastatin Combination for Cardiovascular Events Prevention Trial in Diabetes) assesses efficacy of aspirin, added to simvastatin in patients with either type I or type II diabetes mellitus on development of the primary combined end-point of cardiovascular death, non-fatal myocardial infarction, non-fatal stroke and hospital admission for cardiovascular causes, including acute coronary syndrome, transient ischemic attack, not planned revascularization procedures, peripheral vascular disease [109]. In the ASCEND (A Study of Cardiovascular Events iN Diabetes), patients are randomised to aspirin and/or omega-3 fatty acid for the primary prevention of cardiovascular events [110]. Finally, the TIPS-3 (International Polycap Study 3) trial will assess effect of combination of enteric-coated aspirin and cholecalciferol versus placebo on the composite end point of major cardiovascular disease (cardiac death, non-fatal stroke, non-fatal myocardial infarction), plus heart failure, resuscitated cardiac arrest, or revascularization with evidence of ischemia; aspirin versus placebo on composite of cardiovascular events (cardiac death, myocardial infarction or stroke) and cancer as well as risk of fractures against a cholecalciferol therapy [111]. These new studies would hopefully optimise utilisation of aspirin for primary prevention and identify patients groups who are likely to benefit from such treatment.

#### High On-treatment Platelet Reactivity

High-on-treatment platelet reactivity (or less appropriately termed as aspirin resistance) refers to failure of aspirin to prevent cardiovascular events [112]. It is less related to use of aspirin in hypertension because of limited indications in this group of patients, but representing an important phenomenon of aspirin, platelets and endothelial dysfunction interplay. Such patients were found to have approximately four time higher risk of cardiovascular events, compared to patients with low residual platelet reactivity [113, 114]. It is often explained by insufficient inhibition of TxA_2_ synthesis in platelets; however, precise mechanisms have not been fully elucidated, yet [115].

First, platelets are activated via multiple pathways and receptors, among which aspirin targets only one. Multiple studies showed substantial decrease in TxB_2_ level, reflecting diminished TxA_2_ synthesis; however, applying COX-1 functional testing, as a reliable measure for residual platelet reactivity and prognostication of cardiovascular outcomes, remained controversial [17, 112, 116–119]. Second, non-platelet sources of TxA_2_ generation should be considered, specifically, endothelium and monocytes/macrophages [8, 10, 120]. Therefore, usual once a day dosing regimen may be less effective to assure continuous inhibition of TxA_2_ synthesis [121, 122]. Third, environment in vasculature seems to play important role, e.g. oxidative stress, inflammation, NO synthesis as well as patient characteristics which are causative of the former like as smoking, obesity and diabetes mellitus [9•, 123, 124•, 125•]. For example, brachial flow-mediated dilation was found to be inversely associated with platelet adhesion and aggregation [124•]. Also, immature reticulated platelets were found to be less suppressed by antiplatelet drugs [126].

## Conclusions

Aspirin has been introduced into clinical practice more than a century ago, and its use is supported by large body of evidence, e.g. for secondary prevention of cardiovascular events. Despite this, it is increasingly acknowledged that the multitude of actions of aspirin is not fully elucidated yet. Ongoing trials may spread current use of aspirin to new areas, which are now considered largely controversial. Notwithstanding pleiotropic effects of aspirin on endothelial function, it is unlikely that we will start using aspirin as an antihypertensive agent; however, this may bring additional clinical benefits in selected patients with hypertension, for primary prevention of adverse cardiovascular events.
